# Merging Genomics and Transcriptomics for Predicting Fusarium Head Blight Resistance in Wheat

**DOI:** 10.3390/genes12010114

**Published:** 2021-01-19

**Authors:** Sebastian Michel, Christian Wagner, Tetyana Nosenko, Barbara Steiner, Mina Samad-Zamini, Maria Buerstmayr, Klaus Mayer, Hermann Buerstmayr

**Affiliations:** 1 Institute of Biotechnology in Plant Production (IFA-Tulln), University of Natural Resources and Life Sciences Vienna, 3430 Tulln, Austria; Ch.Wagner1@gmx.at (C.W.); barbara.steiner@boku.ac.at (B.S.); mina.zamini@saatzucht.edelhof.at (M.S.-Z.); maria.buerstmayr@boku.ac.at (M.B.); hermann.buerstmayr@boku.ac.at (H.B.); 2 PGSB Plant Genome and Systems Biology, Helmholtz Center Munich, German Research Center for Environmental Health, 85764 Neuherberg, Germany; tetiana.nosenko@helmholtz-muenchen.de (T.N.); k.mayer@helmholtz-muenchen.de (K.M.); 3 Research Unit Environmental Simulation (EUS) at the Institute of Biochemical Plant Pathology (BIOP), Helmholtz Zentrum München, 85764 Neuherberg, Germany; 4 Saatzucht Edelhof GmbH, 3910 Zwettl, Austria

**Keywords:** wheat, Fusarium head blight, genomic prediction, omics-based prediction, transcriptomics

## Abstract

Genomic selection with genome-wide distributed molecular markers has evolved into a well-implemented tool in many breeding programs during the last decade. The resistance against Fusarium head blight (FHB) in wheat is probably one of the most thoroughly studied systems within this framework. Aside from the genome, other biological strata like the transcriptome have likewise shown some potential in predictive breeding strategies but have not yet been investigated for the FHB-wheat pathosystem. The aims of this study were thus to compare the potential of genomic with transcriptomic prediction, and to assess the merit of blending incomplete transcriptomic with complete genomic data by the single-step method. A substantial advantage of gene expression data over molecular markers has been observed for the prediction of FHB resistance in the studied diversity panel of breeding lines and released cultivars. An increase in prediction ability was likewise found for the single-step predictions, although this can mostly be attributed to an increased accuracy among the RNA-sequenced genotypes. The usage of transcriptomics can thus be seen as a complement to already established predictive breeding pipelines with pedigree and genomic data, particularly when more cost-efficient multiplexing techniques for RNA-sequencing will become more accessible in the future.

## 1. Introduction

The interest in the implementation of predictive breeding for variety development has strongly increased in the plant breeding community during the last decade, and genomic selection with genome-wide distributed molecular markers has become a well implemented tool in many breeding programs [1]. One major target of genomic selection is the genetic improvement of resistance against fungal diseases [2]. Within this framework the resistance of wheat against Fusarium head blight (FHB) is probably one of the most thoroughly studied pathosystems [3]. FHB of wheat is induced by several species among which *Fusarium graminearum*, *Fusarium culmorum*, and *Fusarium sporotrichioides* are the most prominent and agronomically important causal agents conferring large losses in grain yield and contamination of harvested grain with mycotoxins [4]. Since genetic resistance is considered the most environmentally friendly and effective strategy to control diseases, several studies have aimed to increase the achievable gain of predictive breeding for FHB resistance in cereals [5,6,7]. Prediction model improvements range from up-weighting markers linked to major QTL like *Fhb1* [8] to the usage of pre-existing information [9] or traits that are correlated with FHB resistance [10,11]. Anther retention is one promising choice for the latter strategy, since an open-flowering behavior increases plant resistance to initial infection by *Fusarium* spp. [12,13]. 

Besides the genome, other biological strata like the transcriptome and metabolome can serve as an alternative or complement to genomic data in predictive breeding [14,15]. However, the latter two “omics”-methods have not been assessed for predicting FHB resistance in wheat until now [3]. Albeit metabolites can be considered closest to the actual phenotype, and in theory unite the previous biological strata, on the other hand they are also subject to practical constrains like fast turn-over rates. An application of metabolic-based predictions revealed a lower accuracy than predictions based on single nucleotide polymorphism (SNP) markers for yield-associated traits in maize [16,17] and barley [18]. In contrast, transcriptomic-based predictions have been reported to perform at least as good or better than genomic prediction in panels of inbred lines and hybrids in maize [19,20]. Metabolic prediction of hybrid performance in rice was on the other hand competitive to both genomic and transcriptomic prediction [21,22], highlighting the potential of the different biological strata as inputs in a predictive breeding framework. 

Usage of RNA-sequencing data for making predictions has furthermore been hypothesized to tackle biological epistasis [23], which is difficult to address by current statistical models with genome-wide distributed SNP markers [24]. RNA-sequencing is furthermore representative of the expression of actual genes instead of linked loci, and has the potential to serve as an alternative to current genotyping technologies as the underlying data can likewise be used to derive SNP markers and presence/absence variations [25]. Nevertheless, obtaining RNA-sequencing data is currently more costly than obtaining DNA fingerprints with a couple of thousand molecular markers, which renders the merging of incomplete transcriptomic and complete genomic data an interesting option [26]. An analogous problem can be found in animal breeding, where genotyping of hundreds of thousands of animals can be likewise cost restrictive. Blending of incomplete genomic with complete pedigree information in a common relationship matrix by the single-step method is therefore a well-established method in livestock improvement programs [27]. The goals of this study were thus (i) to compare the potential of genomic with transcriptomic prediction using different combinations of gene expressions matrices, and (ii) to assess the merit of blending incomplete transcriptomic with complete genomic data for predicting FHB resistance in winter wheat.

## 2. Materials and Methods

### 2.1. Plant Material

A diverse winter wheat (*Triticum aestivum* L.) panel consisting of 96 genotypes including inbred experimental lines from the Department of Agrobiotechnology IFA-Tulln, advanced generation breeding lines developed in the programs of Florimond Desprez and RAGT as well as further registered cultivars from Austria, Germany, and France was analyzed in this study. Based on SNP Array data (see below) the degree of heterozygosity was in the range of 0.2–7.8% with a median of 1%. The panel was grown in an unbalanced series of field trials at the experimental station of IFA-Tulln (48.31 N, 16.07 E, 180 m elevation, chernozem soil type) in double rows of 90 cm length with a row spacing of 33 cm. The experimental field was separated into distinct trials within each year to facilitate an inoculation with the three different *Fusarium* species: *F. graminearum*, *F. culmorum*, and *F. sporotrichioides*, which vary both in their aggressiveness and mycotoxin spectrum. Each trial was laid out as a randomized complete block design with 2–4 replicates during the years 2011–2015 (Table 1), resulting in an average number of twelve replications of each genotype.

The inoculum was produced as described in [28]. The inoculation started when 50% of the wheat heads in the first plots began to flower (BBCH 65)—this process was repeated every two days until all plots had passed the anthesis stage. All plots were spray-inoculated using a battery-driven backpack sprayer in the late afternoon, and a mist irrigation system provided a high level of humidity to ensure an optimal fungal growth. The FHB severity was scored at 10, 14, 18, 22, and 26 days after anthesis as the percentage of *Fusarium*-infected spikelets per plot, and finally expressed as the Area Under the Disease Progress Curve (AUDPC). Aside from FHB severity, anthesis date was recorded as days after May 1 when 50% of the heads within a plot reached the flowering stage (BBCH 65). In addition, anther retention was assessed by manually opening 20 florets of five randomly chosen heads and expressed as the percentage of florets with at least one retained anther within each double row. The average plant height within each double row was measured in centimeters in all trials at the end of the cropping season (BBCH 89–92).

### 2.2. Statistical Analysis of the Phenotypic Data

Best linear unbiased predictions (BLUP) were derived for each line and trait by analyzing the collected phenotypic data with a linear mixed model of the form:

(1) $$y_{\mathrm{ijkl}}=µ+g_{i}+y_{j}+{\mathrm{yt}}_{\mathrm{jk}}+{\mathrm{ytb}}_{\mathrm{jkl}}+{\mathrm{gy}}_{\mathrm{ij}}+{\mathrm{gyt}}_{\mathrm{ijk}}+e_{\mathrm{ijkl}}$$

where $y_{\mathrm{ijkl}}$ is the phenotypic observation for each trait, µ is the grand mean, and $g_{i}$ is the random effect of the ith line. The effect of the jth year ($y_{j}$) was fixed, while the effect of the kth trial within the jth year (${\mathrm{yt}}_{\mathrm{jk}}$), and the lth block within the kth trial and jth year (${\mathrm{ytb}}_{\mathrm{jkl}}$) were modeled as random. The genotype-by-year interaction (${\mathrm{gy}}_{\mathrm{ij}}$) and genotype- by-year-by-trial interaction (${\mathrm{gyt}}_{\mathrm{ijk}}$) were modeled as random. The residuals followed $e_{k}\sim N\left(0,\sigma_{e_{k}}^{2}\right)$ with a different variance for each of the trials. The same model (1) was used to derive BLUPs for anther retention from all trial-by-year combinations inoculated with two of the three isolates in order to avoid an upward bias of the prediction accuracy when using this trait for indirect phenotypic or trait-assisted omics-based prediction for the individual *Fusarium* species [29]. Specific trial series inoculated with one of the individual isolates were analyzed with the mixed model:

(2) $$y_{\mathrm{ijl}}=µ+g_{i}+y_{j}+{\mathrm{yb}}_{\mathrm{jl}}+{\mathrm{gy}}_{\mathrm{ij}}+e_{\mathrm{ijl}}$$

to derive BLUPs for each genotype with respect to their FHB resistance against *F. graminearum*, *F. culmorum*, and *F. sporotrichioides*. The trial effect was dropped in comparison to the previous model (1) as each isolate was only tested in one trial in per year. Hence, $y_{\mathrm{ikl}}$ designates the phenotypic observations for FHB severity, µ is the grand mean, and $g_{i}$ the random effect of the ith line, $y_{j}$ the effect of the jth year, and ${\mathrm{yb}}_{\mathrm{jl}}$ the effect of the lth block within the jth year. Heterogeneous residual variances were again modeled in (2), although in the case of the trial series with individual isolates each year received its own residual variance. The entry-mean heritability for each trait was determined by

(3) $$h^{2}=1-\frac{{\bar{\mathrm{vd}}}_{\mathrm{BLUP}}}{2\sigma_{g}^{2}}$$

where $\sigma_{g}^{2}$ is the genetic variance and ${\bar{\mathrm{vd}}}_{\mathrm{BLUP}}$ the mean variance of a difference of two genotypic BLUPs [30]. All phenotypic analyses were conducted with the mixed model package *sommer* [31] for R [32].

### 2.3. Genotypic Data and Transcriptome Profiling

All winter wheat lines were genotyped with the TaBW280K SNP array [33], where markers were coded as +1 or −1 for homozygous or 0 for heterozygous allele calls. Markers that were monomorphic or had a missing rate of more than 10% were discarded, while the other missing marker scores were chromosome-wise imputed using the *missForest* algorithm [34]. This resulted in a final set of 10,084 SNP markers, which were subsequently utilized to conduct a principal component analysis for the investigated panel of 96 genotypes (Supplementary Figure S1).

For the purpose of RNA-sequencing, all genotypes were grown under controlled greenhouse conditions as described by [35]. One water-inoculated i.e., mock-treated and two *Fusarium*-inoculated replicates were planted in the greenhouse in a randomized complete block design with 10 plants per pot. Six individual heads per pot were inoculated at mid-anthesis (BBCH 65); the basal florets of their central spikelets were point inoculated by pipetting 10 µL of either a *F. graminearum* spore suspension (strain IFA65/66; 50,000 spores mL^−1^) or distilled water between palea and lemma. The wheat heads were subsequently covered with plastic bags for 24 hours to ensure a high humidity for an optimal fungal growth. The inoculations were repeated in the morning of consecutive days to minimize possible confounding effects of daytime dependencies i.e., circadian gene expressions. The six treated heads per pot were harvested 48 h after inoculation, and the treated spikelet separated into lemma, palea, and rachis on one side and the reproductive tissues on the other side. After excluding awns, the sampled lemma, palea, and rachis tissue was immediately shock-frozen in liquid nitrogen and stored at −80 °C for RNA extraction. RNA was extracted from 100 mg of pooled plant material from five to six heads harvested from each individual pot using the RNeasy Plant Mini Kit from Qiagen (Hilden, Germany) following the manufacturer’s instructions. 

RNA-sequencing was done by GATC Biotech (Konstanz, Germany) using Illumina HiSeq 2500 strand-specific sequencing technology with the 125 bp paired-end mode for the 288 libraries that corresponded to the individual samples from the 96 genotypes harvested from the two times replicated *Fusarium*-treatment and the mock-treatment respectively. Adapters and low-quality reads were trimmed using *Trimmomatic v.0.35* [36], while the relative content of *Fusarium* and wheat reads in each RNA-sequencing library was estimated by *FastQ Screen* [37]. The processed RNA-sequencing data were aligned to the *T. aestivum* L. IWGSC v1.0 reference genome sequence [38] using *Hisat2 v. 2.1.0* [39], and reads that aligned genes to a single locus were counted with the *featureCounts* software [40]. A feature matrix of variance stabilized counts adjusted for the library size was subsequently obtained by using the R package *DESeq2* [41]. A set of 90,093 expressed genes represented by at least 10 normalized counts in at least five RNA-seq libraries was used as an input for fitting transcriptomic-based prediction models. 

### 2.4. Single-Trait Omics-Based Prediction

The potential to predict FHB resistance, anther retention, plant height, and anthesis date across all isolates using genomic and transcriptomic predictor variables was assessed by conducting a leave-one-out cross-validation and fitting single-trait best linear unbiased prediction (BLUP) models for each of these traits:

(4) $$y_{i}=µ+g_{i}+e_{i}$$

where µ is the grand mean, $y_{i}$ is the BLUP of the ith genotype obtained in the phenotypic data analysis, and $e_{i}$ the residual effect with $e\sim N\left(0,I\sigma_{e}^{2}\right)$. The effect $g_{i}$ of the ith genotype was modelled as random to derive additive genotypic effects with $g\sim N\left(0,G\sigma_{g}^{2}\right)$ by genomic best linear unbiased prediction (GBLUP), where the genomic relationship matrix G was computed following [42]:

(5) $$G=WW^{T}/2\Sigma \left(1-p_{l}\right)p_{l}$$

where W is a centred marker matrix of the j lines with $W_{\mathrm{jl}}=Z_{\mathrm{jl}}+1-2p_{l}$ and $p_{l}$ is the allele frequency of the +1 allele at the lth locus. Transcriptomic best linear unbiased predictions (TBLUP) were analogously derived by modelling $g\sim N\left(0,T\sigma_{g}^{2}\right)$, where the transcriptomic kernel matrix T was obtained from:

(6) $$T=MM^{T}/f$$

with M being the centred and standardized matrix of expressed genes and f the number of genes. The matrix M itself was built either with gene expression data obtained from the mock-treated samples or one of the *Fusarium*-treated samples. Additionally, all pairwise combinations among them were constructed to investigate the influence of replications in RNA-sequencing experiments on the transcriptomic prediction ability. This combination was achieved by summing across a pair of given gene expression matrices and dividing the result by two before standardizing the combined matrix to derive T in Equation (6). The method was accordingly adjusted when combining the gene expression data from all three treatment-by-replication combinations:

(7) Mcombi=13(Mfhb[rep1]+Mfhb[rep2]+Mmock)

where the combined matrix $M_{\mathrm{combi}}$ was derived from the average of the gene expression matrices $M_{\mathrm{fhb}\left[\mathrm{rep}1\right]}$ and $M_{\mathrm{fhb}\left[\mathrm{rep}2\right]}$ as well as $M_{\mathrm{mock}}$ representing the two replicates of the *Fusarium*-treatment and the mock-treatment, respectively. Using simple means across the different matrices was equivalent to obtaining best linear unbiased estimates of the transcriptomic predictors as was done by [23], since the RNA-sequencing experiment was completely balanced in the study at hand. Aside from comparing molecular markers and gene expression data obtained from different treatments, we examined the impact of the predictor number on the prediction ability by randomly sampling sets between 500 to 80,000 genomic and transcriptomic predictors for generating the matrices G and T. The prediction ability of all models was assessed by correlating the predicted performance values with the observed values derived in the phenotypic data analysis. All models for genomic and transcriptomic prediction were fitted with *sommer* [31] for the R statistical environment [32].

### 2.5. Trait-Assisted and Single-Step Prediction Models

The above-described genomic and transcriptomic single-trait prediction models were subsequently extended by including anther retention as a covariate to test the potential of increasing the prediction ability for FHB resistance in a trait-assisted prediction model of the form:

(8) $$y_{i}=µ+\gamma \cdot x_{i}+g_{i}+e_{i}$$

where µ is the grand mean, $g_{i}$ is the random effect of the of the ith line, and $y_{i}$ is the FHB resistance against either *F. sporotrichioides*, *F. culmorum,* or *F. graminearum* that were regarded separately in this case. The covariate $x_{i}$ (anther retention) was for the purpose of trait-assisted prediction measured in trials that differed from the ones in which the trait of interest was assessed to avoid an upward bias of the prediction ability [29]. The estimated breeding value of the ith line was thus computed by also taking the regression coefficient γ into account

(9) $${\mathrm{EBV}}_{i}=µ+\hat{\gamma }\cdot x_{i}+{\hat{g}}_{i}$$

where $\hat{\gamma }$ and ${\hat{g}}_{i}$ are the estimate of the regression coefficient and the predicted genotype performance respectively, and $x_{i}$ is in this case the observed phenotype for anther retention of the ith genotype. Like for the single-trait predictions a leave-one-out cross-validation was conducted to assess both the genomic and transcriptomic prediction ability.

This evaluation was further extended to the single-step framework to combine both information sources when predicting the resistance against the individual FHB isolates. Since generating transcriptomic data is currently more expensive than obtaining genomic fingerprints, the percentage of RNA-sequenced genotypes was varied in the range of 10–90% when fitting the single-step genomic-transcriptomic prediction (ssGTBLUP) models. The entire panel was 100 times randomly split into sets of genotypes for which only genomic information were available and sets with both genomic and transcriptomic information. Additionally, a partitioning around medoids clustering [43] was conducted to more directly test the merit of sampling a diverse set of genotypes for the more costly RNA-sequencing with the *pamk* function from the *fpc* R package [44]. This was facilitated by varying cluster number in the range 9–86, i.e., an equivalent of approximately 10–90% of the entire panel, and sampling the lines representing the medoid of each cluster into the set of RNA-sequenced lines. The method aimed thus to uniformly cover the given target genetic space with these lines in a similar way as [45].

Both models (4) and (8) were afterwards fitted with the assumption of $g\sim N\left(0,H_{GT}\sigma_{g}^{2}\right)$, which was compared with predictions based solely on genomic or transcriptomic data using $g\sim N\left(0,G\sigma_{g}^{2}\right)$ and $g\sim N\left(0,T\sigma_{g}^{2}\right)$ respectively. The hybrid relationship matrix $H_{GT}$ for merging genomic and transcriptomic information was obtained by modifying the method suggested by [46] and [47] to:

(10) $$H_{GT}=\left(\begin{array}{cc} G_{11}-G_{12}G_{22}^{-1}\left(T-G_{22}\right)G_{22}^{-1}G_{21} & G_{12}G_{22}^{-1}T\\ TG_{22}^{-1}G_{21} & T \end{array}\right).$$

where the matrix $G_{11}$ contains the genomic relationship between non-RNA-sequenced genotypes, $G_{22}$ the genomic relationship between RNA-sequenced genotypes, while $G_{12}$ and $G_{21}$ model the genomic relationship between RNA-sequenced and non-RNA-sequenced genotypes. The prediction abilities of all genomic and transcriptomic models were again assessed by correlating the predicted performance values with the observed values derived in the phenotypic analysis of the data. The prediction ability for an indirect phenotypic selection for FHB resistance by anther retention was obtained by correlating the observed values of anther retention with the observed values of FHB resistance against the individual isolates. All trait-assisted and single-step prediction models were fitted with *sommer* [31], and the hybrid relationship matrices $H_{GT}$ were generated with the R package *AGHmatrix* [48].

## 3. Results

A large variation and high entry-mean heritability was observed for all investigated traits including FHB resistance (Table 2). The correlation between FHB resistance and anthesis date was low (r = 0.10) (Supplementary Figure S2). However, a strong correlation between FHB and plant height (r = −0.57) as well as between anther retention and plant height (r = −0.55) could be observed. A high and significant correlation was furthermore seen between FHB severity and anther retention (r = 0.69). Even when adjusting FHB severity and anther retention by plant height using the residual method [49] a high to moderate correlation of r = 0.55 between the two former traits was retained, underlining the potential of anther retention as a phenotypic predictor of FHB severity without an unfavorable trade-off with respect to the selection of comparably tall plants.

**Figure 1:**
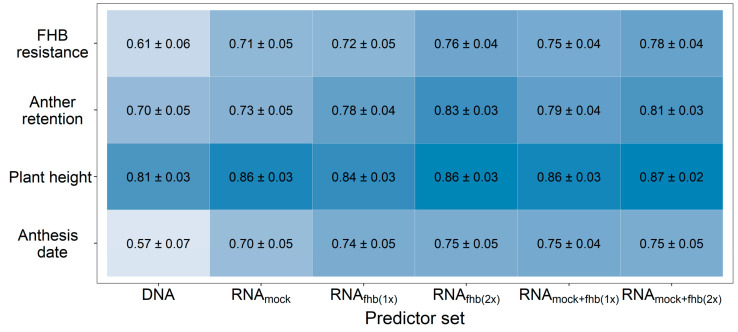
Prediction ability for FHB resistance, anther retention, plant height, and anthesis date using different predictor sets for a genomic prediction with 10,084 SNP markers (DNA) or 90,093 expressed genes (RNA). The usage of individual feature matrices of the transcriptomic data obtained from mock-treated plants (RNA_mock_) or *Fusarium*-treated plants (RNA_fhb(1x)_) was compared with a combination from both *Fusarium*-treated replications (RNA_fhb(2x)_) or from one mock-treated and one *Fusarium*-treated replicate at time (RNA_mock+fhb(1x)_) as well as the integration of gene expression data from all treatment-by-replication combinations (RNA_mock+fhb(2x)_). Darker coloring indicates a higher average prediction ability.

The usage of genomic prediction likewise showed some potential for the selection of FHB resistant genotypes with a high prediction ability of r = 0.61, which was increased up to r = 0.76 by using transcriptomic data for fitting prediction models (Figure 1). Analogous observations were made for the other investigated traits, while the highest merit other than for FHB was observed for the anthesis date. The lowest merit was seen in the highly heritable trait, plant height. A very similar prediction ability was achieved by using transcriptome data from either the *Fusarium*-treated or mock-treated plants for predicting FHB resistance. Combining different transcriptomic feature matrices from these treatments into a single kernel was generally more advantageous than utilizing RNA-sequencing data obtained from a single treatment-by-replicate combination.

**Figure 2:**
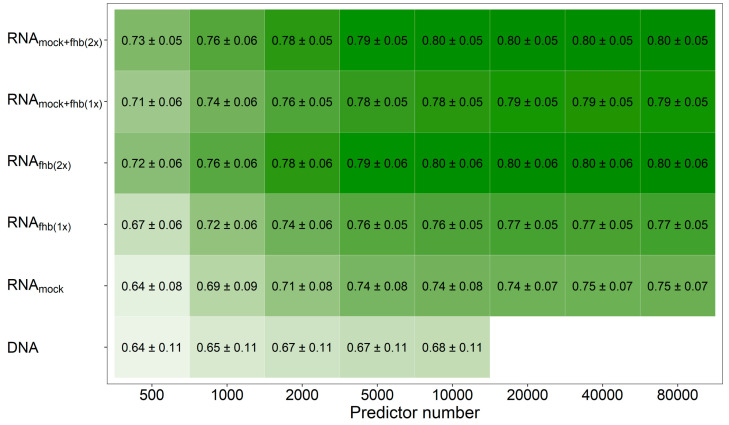
Average prediction ability using an increasing number of genomic and transcriptomic predictors. The merit of molecular markers (DNA) for fitting prediction models was compared with utilizing the individual feature matrices of the transcriptomic data obtained from mock-treated plants (RNA_mock_) or *Fusarium*-treated plants (RNA_fhb(1x)_), a combination from both *Fusarium*-treated replications (RNA_fhb(2x)_) or from one mock-treated and one *Fusarium*-treated replicate at time (RNA_mock+fhb(1x)_) as well as an integration of gene expression data from all treatment-by-replication combinations (RNA_mock+fhb(2x)_). Darker coloring indicates a higher average prediction ability.

Mixing all three feature matrices together revealed, averaged across all traits, a marginal benefit (r = 0.81) in comparison to the pairwise combination of feature matrices based either on the two *Fusarium*-treatments (r = 0.80) or the mock-treatment and one *Fusarium*-treatment at a time (r = 0.79). This observation was also made when varying the number of the employed predictors that were used to generate these relationship matrices, where the transcriptomic data showed a general advantage over molecular markers at same number of predictors if at least two feature matrices were combined (Figure 2; Supplementary Figure S3). Averaged across all traits, a number of 5000–10,000 molecular markers were thus required to achieve the same prediction ability as with the information given by 500–1000 expressed genes.

**Figure 3:**
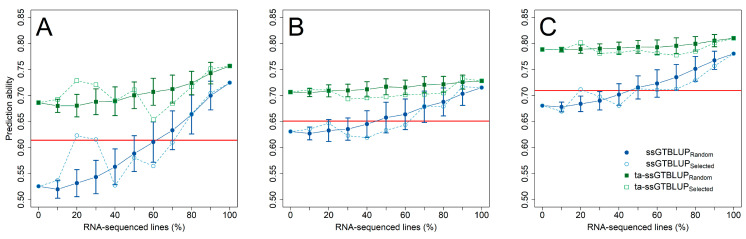
Prediction ability (±standard deviation) for the resistance against *F. sporotrichioides* (**A**), *F. culmorum* (**B**), and *F. graminearum* (**C**) with an increasing number of RNA-sequenced genotypes for a single-trait single-step genomic-transcriptomic prediction (ssGTBLUP) (blue circles) and a trait-assisted single-step genomic-transcriptomic prediction (ta-ssGTBLUP) (green squares) that includes pre-existing information of the secondary trait anther retention. The set of RNA-sequenced genotypes was either randomly sampled (closed symbols; solid lines) or by the partitioning around medoids method based on SNP Array markers (DNA) (open symbols; dashed lines). Single-step predictions without RNA-sequenced genotypes correspond to genomic prediction and models with a complete set of RNA-sequenced genotypes to transcriptomic predictions. Prediction models were compared with the merit of an indirect phenotypic selection based on anther retention (solid red horizontal line). All three gene expression matrices were used to fit the single-step and transcriptomic prediction models.

**Figure 4:**
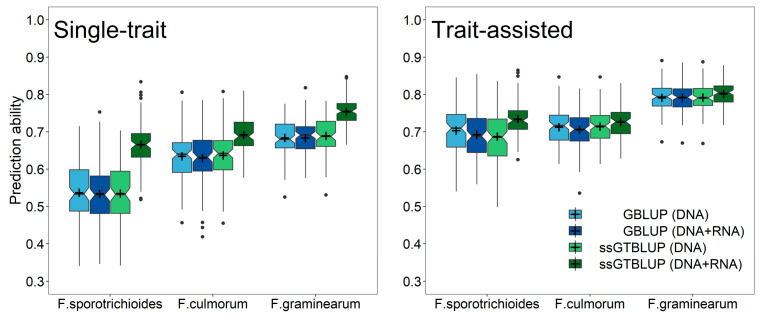
Prediction ability for resistance against three tested *Fusarium* isolates using single-step genomic-transcriptomic prediction (ssGTBLUP) within the groups of RNA-sequenced (dark green) and non-RNA-sequenced genotypes (light green) as well as genomic prediction (GBLUP) within the group of RNA-sequenced (dark blue) and non-RNA-sequenced genotypes (light blue). Result are shown for the single-trait predictions (left) and the trait-assisted prediction with exploiting pre-existing information about anther retention (right). The presented results are based on a split with half of the genotypes being part of RNA-sequenced and the other half of the non-RNA-sequenced group.

This advantage of gene expression data over SNP markers was likewise realized in the prediction of the individual *Fusarium* isolates (Figure 3), while at least 50–60% of the genotypes had to be RNA-sequenced in the single-step predictions with incomplete transcriptomic data before performing equally well to an indirect phenotypic selection by anther retention. Nevertheless, exploiting pre-existing information of anther retention in trait-assisted single-step genomic-transcriptomic prediction models always resulted in highest prediction ability irrespective of the employed transcriptomic matrices (Supplementary Figure S4). The advantage of increasing the number of RNA-sequenced genotypes was lower in these cases, while choosing a diversity set by a training population design algorithm did not reveal a clear additional gain in prediction ability. The increase in prediction ability in the single-step predictions was mostly attributed to an increased accuracy among the RNA-sequenced genotypes, while the ranking among the non-RNA-sequenced genotypes was similar to the ranking in a genomic prediction using only SNP markers (Figure 4; Supplementary Figure S5). This advantage within the group of RNA-sequenced genotypes diminished though in the trait-assisted prediction with the secondary trait anther retention, which corroborates the previously mentioned observation in the predictions across the entire set of RNA-sequenced and non-RNA-sequenced genotypes.

## 4. Discussion

A fundamental question when using transcription data for making predictions is the choice of an appropriate time-point [35,50] and tissue for RNA sampling [25], since transcriptome analyses are only reflecting snapshots of the concerted gene expression. Nevertheless, samples for transcriptomic-based predictions were taken from seeds [19], whole-seedlings [51,52], flag leaves of adult plants [22], or from ears/heads as in the study at hand. The potential targets of such predictions are ranging from characteristics assessed in the seedling stage, e.g., juvenile growth to agronomic traits measured on mature plants like grain yield. It might thus be hypothesized that the relative high performance of the transcriptome for predicting FHB resistance, which is expressed in the generative phase of plants, is partially explained by a sampling that was likewise conducted at this phase in the greenhouse.

Transcriptomic data are often based on samples taken under controlled greenhouse conditions; thus, transferability of such snapshots of gene expression for the purpose of predictions made in different environments is not self-evident. Although RNA can be regarded as being closer to the actual phenotype in comparison to DNA, the former is also more prone to noise during sampling [17] and furthermore subject to gene-by-environment interaction caused by varying abiotic [53] and biotic sampling conditions [35]. Despite such interactions, very similar prediction abilities for FHB resistance (Δ = 1.4%) were found by utilizing feature matrices based either on *Fusarium*-treated or mock-treated samples. Differential gene expression analysis showed that approximately 10% of the genes were differentially expressed between the *Fusarium*-treatment and mock-treatment [54]. From this group of genes only 20% (=2% of all expressed genes) were differentially expressed between groups of resistant, moderate resistant, and susceptible genotypes. Another 5% among all expressed genes were on the other hand consecutively expressed between these resistance groups. The concurrent presence of differential gene expression information for this larger consecutively expressed gene subset in the RNA-sequencing data from *Fusarium*-treated as well as mock-treated wheat plants might thus explain the small difference in prediction ability of FHB resistance with the alternative feature matrices.

The average correlation among the magnitude of the expression from the individual genes between the mock-treatment and *Fusarium*-treatments was in the range of r = 0.33–0.34. Although this association was slightly higher (r = 0.39) between two replicates of the *Fusarium*-treatment, it suggested a low-to-moderate repeatability of RNA-sequencing data without biological replicates. A combination of feature matrices from both biological replicates of the *Fusarium*-treatment into a common matrix was promising since the average reliability of the predictors increased from the estimate of h²_rep_ = 0.36 to h²_rep_ = 0.52. This gain was furthermore higher than the added noise even when averaging across the mock- and *Fusarium*-treatments resulting in a repeatability of h²_rep_ = 0.54. Increasing the number of biological replicates has also been reported to have a larger impact than library size on the power of differential gene expression and ontology enrichment analysis [55]. A transcriptomic-based prediction employing unreplicated gene expression data showed nonetheless an advantage over a genomic-based prediction in the study at hand. This might be to some extent explained by theoretical factors like addressing biological epistasis by RNA-sequencing data as well as a larger number of predictors in transcriptomic predictions. Augmenting the transcriptomic data with a higher number of biological replicates increased this difference further, even at the same number of predictors.

However, as costs are likewise higher for RNA-sequencing than DNA-fingerprinting, and this cost difference increases with each additional library, a more cost-efficient option might be given by obtaining gene expression data only from a subset of genotypes in combination with complete SNP marker data in a given set of genotypes. Exploiting the single-step methodology developed in animal breeding, [26] showed the potential of this strategy for predicting major agronomic traits in hybrid maize using either pedigree or genomic in combination with the aforementioned transcriptomic data. Increasing the number of RNA-sequenced lines in transcriptomic-based single-step prediction resulted likewise in a higher prediction ability for FHB resistance in comparison to genomic prediction, which was however merely marginally higher when integrating pre-existing information of the correlated trait anther retention into the models. Regarding the small advantage for non-RNA-sequenced genotypes in single-step genomic-transcriptomic predictions, it might thus be argued that its merit is probably largest in scenarios where pre-existing information for the array of agronomically relevant traits is absent. An implementation of transcriptomics into a predictive breeding framework seems currently feasible in applied breeding programs via sets of few pre-selected parents in order to support in an omics-assisted planning of crosses, especially when crossing earlier and if pre-existing agronomic information of potential parents is only partially available. Such a strategy could, in a broader context, also increase the across-family prediction ability for the larger population of progenies derived from these parents when performance information is not yet available in early breeding generations, and thus extend upon existing pedigree-based and genomic-based approaches. 

## 5. Conclusions

Implementing transcriptomics into the predictive breeding framework has a potential to obtain more accurate predictions. The current cost-restrictions are making this endeavor mostly realizable in the single-step prediction framework or other methods for integrating multiple kernels. The use of transcriptomics can thus be seen as a complement to already established pipelines with pedigree and genomic data. This is particularly valid when more cost-efficient multiplexing techniques for RNA-sequencing [56,57] will become more accessible in the future.

## Figures and Tables

**Table 1:** Replicates (in brackets) and number of lines tested from the complete set of 96 lines within each trial inoculated with *F. sporotrichioides* (FS), *F. culmorum* (FC), or *F. graminearum* (FG) between 2011 and 2015. The smaller subset of lines tested in 2011 was extended by adding another set of 32 lines in 2012, which was finally extended by further 38 lines in 2013–2015.

	Trial		
Year	FS	FC	FG
2011	26 (2)		26 (4)
2012	58 (2)		58 (2)
2013	96 (2)		96 (2)
2014		96 (2)	
2015	96 (2)	96 (2)	

**Table 2:** Mean, range, variance components, and heritability for Fusarium head blight (FHB) severity (AUDPC), anther retention (AR) (%), plant height (PH) (cm), and anthesis date (AD) (days after May 1st) for the phenotypic analyses across all trials, trials inoculated with one of the *F. sporotrichioides*, *F. culmorum*, and *F. graminearum* isolates, respectively, as well as excluding one of these trial series at a time.

Set	Trait	Min	Mean	Max	$\sigma_{g}^{2}$	$\sigma_{gy}^{2}$	$\sigma_{gyi}^{2}$	$\sigma_{e}^{2}$	$H^{2}$	$h^{2}$
All trials	FHB	51.89	114.62	282.47	2338.49	0.00	629.45	10226.20	0.18	0.94
	AR	0.00	52.96	93.13	734.03	68.81	22.42	156.29	0.75	0.97
	PH	65.71	78.23	104.68	72.36	3.74	0.00	10.84	0.83	0.98
	AD	20.20	24.67	30.00	5.62	0.85	0.00	1.36	0.72	0.97
Isolates ^†^	FHB_FS_	0.00	39.40	177.92	1308.28	335.18		831.78	0.53	0.91
	FHB_FC_	17.04	227.36	846.55	35605.89	3776.73		6610.13	0.77	0.95
	FHB_FG_	69.34	201.10	452.40	7572.85	1877.78		5436.90	0.51	0.87
Ind. Sel. ^‡^	AR_woFS_	0.00	50.07	91.55	744.94	47.46	47.46	160.38	0.74	0.97
	AR_woFC_	0.00	50.55	84.34	568.15	68.44	24.75	174.89	0.68	0.93
	AR_woFG_	0.00	54.45	93.73	782.06	42.69	42.69	129.45	0.78	0.97

Genotypic variance ($\sigma_{g}^{2}$), genotype-by-year interaction variance ($\sigma_{\mathrm{gy}}^{2}$), genotype-by-year-by-isolate interaction variance ($\sigma_{\mathrm{gyi}}^{2}$), averaged residual variance ($\sigma_{e}^{2}$), and plot-based ($H^{2}$) and entry-mean heritability ($h^{2}$). ^†^ FHB severity for the trial series inoculated with *F. sporotrichioides* (FS), *F. culmorum* (FC), and *F. graminearum* (FG). ^‡^ Anther retention across trials, excluding trials inoculated either with *F. sporotrichioides* (woFS), *F. culmorum* (woFC), or *F. graminearum* (woFG) to assess the merit of an indirect phenotypic and a trait-assisted omics-based prediction.

## Data Availability

Data is contained within the article and as supplementary material.

## Supplementary Materials

The following are available online at https://www.mdpi.com/2073-4425/12/1/114/s1, Supplementary Figure S1: Principal component analysis of the 96 genotypes from IFA-Tulln, RAGT, Florimond Desprez as well as the set of registered varieties involved in the study using either the SNP markers (DNA), RNA-sequencing data from the mock-treatment (RNA_mock_) or the Fusarium-treated plant samples from the first (RNA_fhb(rep1)_) or second replicate (RNA_fhb(rep2)_) in the greenhouse; Supplementary Figure S2: Phenotypic correlation between FHB severity (AUDPC), anther retention (AR) (%), plant height (PH) (cm), and anthesis date (AD) (days after May 1^−*+^) for the phenotypic analyses across all trials; Supplementary Figure S3. Prediction ability (±standard deviation) for FHB resistance (A), anther retention (B), plant height (C), and anthesis date (D) using an increasing number of genomic and transcriptomic predictors; Supplementary Figure S4. Prediction ability (±standard deviation) for the resistance against *F. sporotrichioides* (A), *F. culmorum* (B), and *F. graminearum* (C) with an increasing number of RNA-sequenced genotypes for a single-trait single-step genomic-transcriptomic prediction (ssGTBLUP) (blue circles) and a trait assisted single-step genomic-transcriptomic prediction (ta-ssGTBLUP) (green squares) that includes pre-existing information of the secondary trait anther retention; Supplementary Figure S5. Prediction ability for resistance against the three tested FHB isolates using single-step genomic-transcriptomic prediction (ssGTBLUP) within the group of RNA-sequenced (dark green) or non-RNA-sequenced genotypes (light green) as well as genomic prediction (GBLUP) within the group of RNA-sequenced (dark blue) or non-RNA-sequenced genotypes (light blue).
