# Supplementary material for: Merging Genomics and Transcriptomics for Predicting Fusarium Head Blight Resistance in Wheat
Source: Genes (Basel). 2021 Jan 19;12(1):114. doi: 10.3390/genes12010114 (PMC7832326; doi:10.3390/genes12010114)
Supplement: Supplementary file 1 [file genes-12-00114-s001.zip › Supplementary material GS RNAseq.pdf]

Supplementary material

Article Title: Merging genomics and transcriptomics for predicting Fusarium head blight resistance in winter wheat

Journal: Genes (ISSN 2073-4425; CODEN: GENEG9)

Authors: Sebastian Michel, Christian Wagner, Tetyana Nosenko, Barbara Steiner, Mina Samad-Zamini, Maria Buerstmayr, Klaus Mayer, Hermann Buerstmayr

Name, affiliation, and email of corresponding author:

Sebastian Michel
Department for Agrobiotechnology (IFA-Tulln)
Institute for Biotechnology in Plant Production
University of Natural Resources and Life Sciences, Vienna (BOKU)
Konrad-Lorenz-Str. 20, 3430 Tulln, Austria
e-mail: sebastian.michel@boku.ac.at

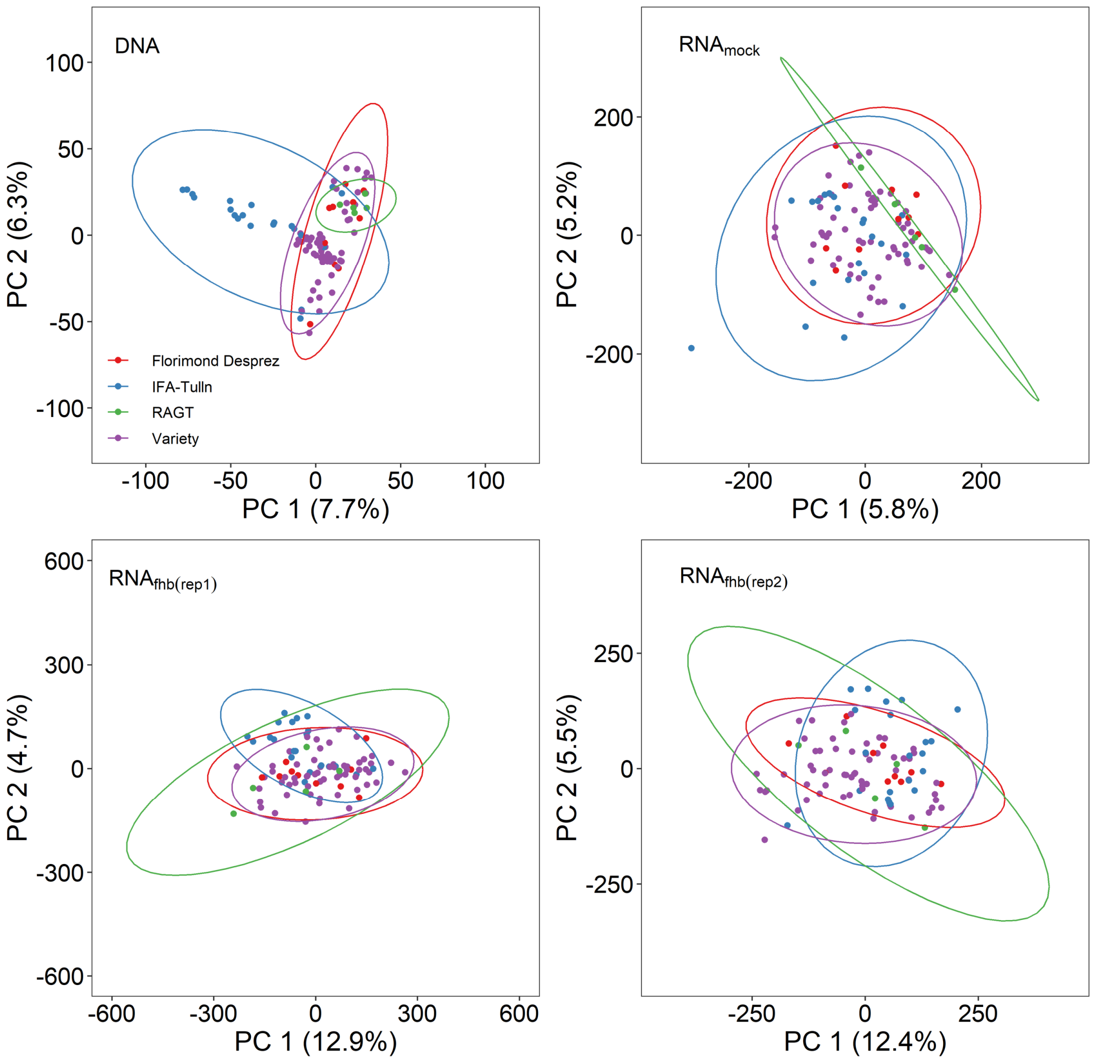

Supplementary Figure S1. Principal component analysis of the 96 genotypes from IFA-Tulln, RAGT, Florimond Desprez as well as the set of registered varieties involved in the study using either the SNP markers (DNA), RNA-sequencing data from the mock-treatment (RNA_{mock}) or the *Fusarium*-treated plant samples from the first ($RNA_{fhb(rep1)}$) or second replicate ($RNA_{fhb(rep2)}$) in the greenhouse.

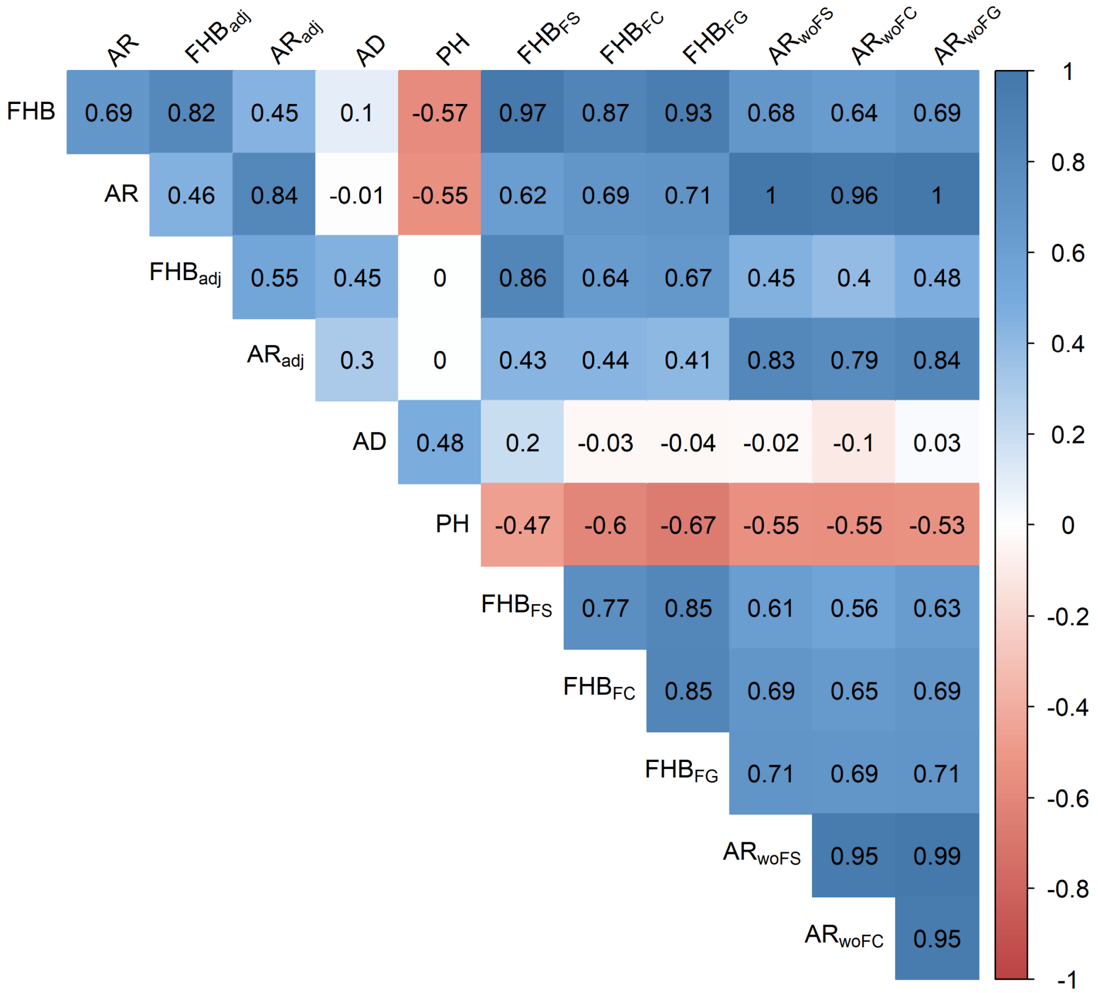

Supplementary Figure S2. Phenotypic correlation between FHB severity (AUDPC), anther retention (AR) (%), plant height (PH) (cm), and anthesis date (AD) (days after May 1st) for the phenotypic analyses across all trials. The correlations with the FHB severity for trials inoculated with one of the *F. sporotrichioides* (FHB$_{FS}$), *F. culmorum* (FHB$_{FC}$), and *F. graminearum* (FHB$_{FG}$) isolates respectively as well as excluding one of these trial series at a time for assessing the merit of an indirect selection by the anther retention are likewise shown (AR$_{woFS}$, AR$_{woFC}$, AR$_{woFG}$). FHB severity (FHB$_{adj}$) and anther retention (AR$_{adj}$) from the across trial analyses were furthermore adjusted by computing the residuals from a linear regression on plant height [1].

1. Hänsel, H. Yield potential of barley corrected for disease infection by regression residuals. *Plant Breed.* **2001**, *120*, 223–226, doi:10.1046/j.1439-0523.2001.00596.x.

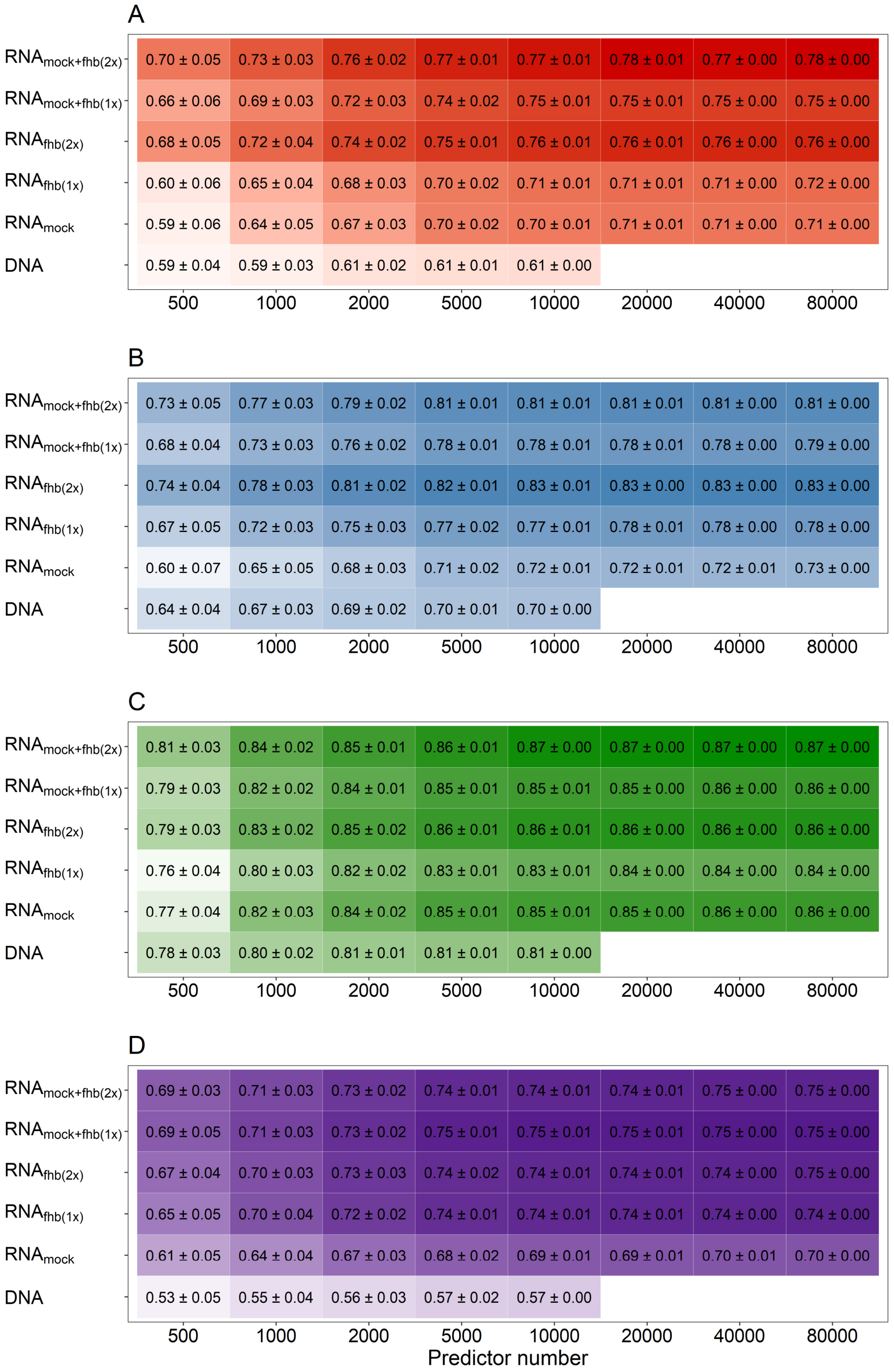

Supplementary Figure S3. Prediction ability (±standard deviation) for FHB resistance (A), anther retention (B), plant height (C), and anthesis date (D) using an increasing number of genomic and transcriptomic predictors. The merit of molecular markers (DNA) for fitting prediction models was compared with utilizing the individual feature matrices of the transcriptomic data obtained from mock-treated plants (RNA_{mock}) or *Fusarium*-treated plants ($RNA_{fhb(1x)}$), a combination from both *Fusarium*-treated replications ($RNA_{fhb(2x)}$) or from one mock-treated and one *Fusarium*-treated replicate at time ($RNA_{mock+fhb(1x)}$) as well as an integration of gene expression data from all treatment-by-replication combinations ($RNA_{mock+fhb(2x)}$).

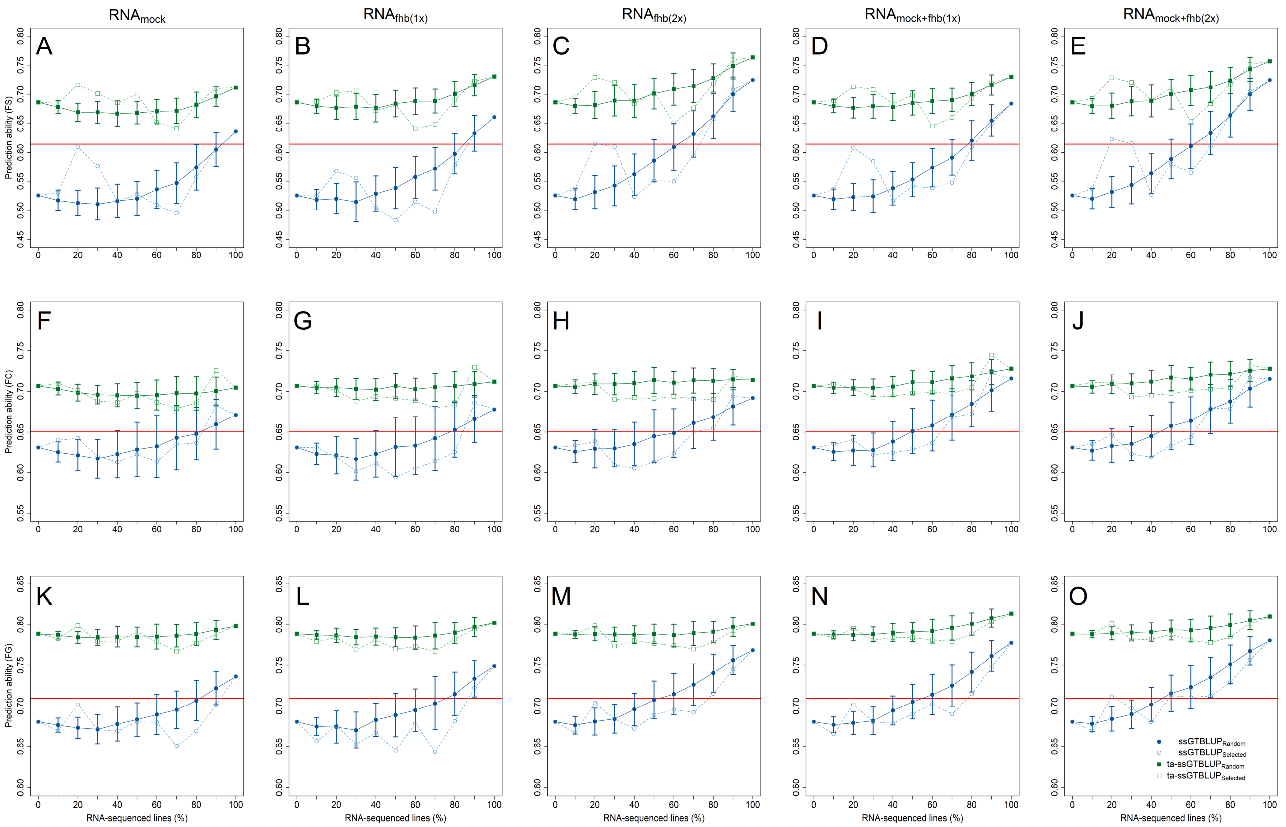

Supplementary Figure S4. Prediction ability (±standard deviation) for the resistance against *F. sporotrichioides* (A), *F. culmorum* (B), and *F. graminearum* (C) with an increasing number of RNA-sequenced genotypes for a single-trait single-step genomic-transcriptomic prediction (ssGTBLUP) (blue circles) and a trait-assisted single-step genomic-transcriptomic prediction (ta-ssGTBLUP) (green squares) that includes pre-existing information of the secondary trait anther retention. The set of RNA-sequenced genotypes was either randomly sampled (closed symbols; solid lines) or by the partitioning around medoids method based on molecular markers (DNA) (open symbols; dashed lines). Single-step predictions without RNA-sequenced genotypes correspond to genomic prediction and models with a complete set of RNA-sequenced genotypes to transcriptomic predictions. Prediction models were compared with the merit of an indirect phenotypic selection based on anther retention (solid red horizontal line). The according prediction models were fitted by employing feature matrices of the transcriptomic data obtained either from water-inoculated i.e. mock-treated plants (RNA_{mock}) or *Fusarium*-treated plants ($RNA_{fhb(1x)}$), a combination from both *Fusarium*-treated replications ($RNA_{fhb(2x)}$) or from one mock-treated and one *Fusarium*-treated replicate at time ($RNA_{mock+fhb(1x)}$) as well as an integration of gene expression data from all treatment-by-replication combinations ($RNA_{mock+fhb(2x)}$).

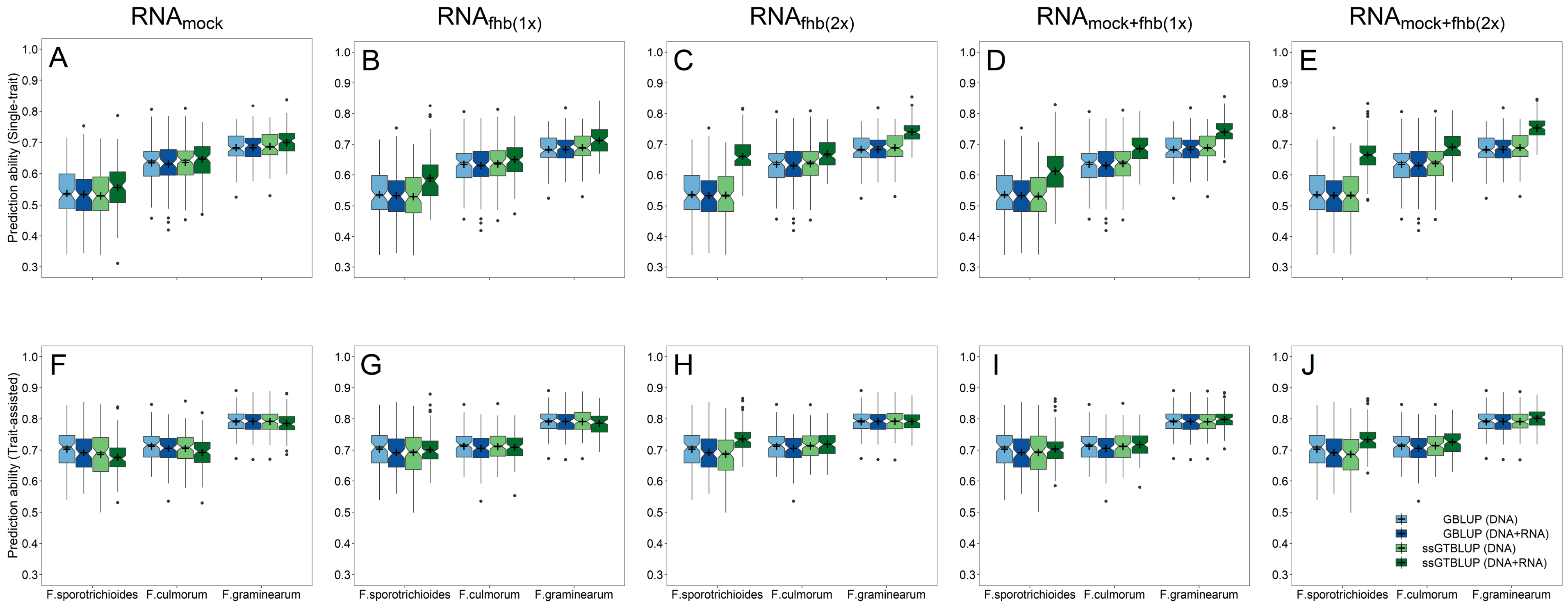

Supplementary Figure S5. Prediction ability for resistance against the three tested FHB isolates using single-step genomic-transcriptomic prediction (ssGTBLUP) within the group of RNA-sequenced (dark green) or non-RNA-sequenced genotypes (light green) as well as genomic prediction (GBLUP) within the group of RNA-sequenced (dark blue) or non-RNA-sequenced genotypes (light blue). Result are shown for the single-trait predictions (A-E) and the trait-assisted prediction with exploiting pre-existing information about anther retention (F-J). The presented results are based on a split with half of the genotypes being part of RNA-sequenced and the other half of the non-RNA-sequenced group. The according prediction models were fitted by employing feature matrices of the transcriptomic data obtained either from mock-treated plants (RNA_{mock}) or *Fusarium*-treated plants ($RNA_{fhb(1x)}$), a combination from both *Fusarium*-treated replications ($RNA_{fhb(2x)}$) or from one mock-treated and one Fusarium-treated replicate at time ($RNA_{mock+fhb(1x)}$) as well as an integration gene expression data from all treatment-by-replication combinations ($RNA_{mock+fhb(2x)}$).
